# Pay to win? Exploring medical students’ use of, and access to, paid commercial educational resources

**DOI:** 10.1186/s12909-025-07233-4

**Published:** 2025-05-21

**Authors:** Michaela Vernon, Nadin Hawwash, Enam Haque, Harish Thampy

**Affiliations:** School of Medical Sciences, Faculty of Biology, Medicine and Health, University of Manchester, Manchester, UK

**Keywords:** Medicine, Widening participation, Student success, Medical students, Assessment

## Abstract

**Background:**

Medical students are often driven by assessment-focused outcomes, aiming not only to pass and progress but also to enhance their academic rankings and achieve awards that influence future recruitment in a highly competitive job market. Consequently, there is growing reliance on the rapidly expanding range of commercial educational resources. However, these resources typically require paid subscriptions, creating barriers for students, particularly those from widening participation backgrounds. This study examines the support medical schools provide for paid commercial educational resources and explores medical students’ perceptions of these resources, with a focus on potential inequities.

**Methods:**

Two separate questionnaires were utilised. The first was distributed to assessment leads at all UK medical schools to determine whether formal guidance or funding for paid resources was provided. The second was sent to all 915 Year 3 and Year 4 students at our medical school to assess their annual expenditure on paid commercial resources, frequency of use, perceived impact on examination outcomes, and attitudes toward the integration of these resources into their studies.

**Results:**

Thirty out of 43 (70%) medical schools completed the first questionnaire. Half provided guidance on paid resources, and seven offered funding support. Schools expressed concern about the quality assurance process in place for commercial paid-for platforms and the risks this could pose for students’ learning. Ninety-nine out of 915 (10.8%) medical students responded to the second questionnaire; 96% reported paying for online resources, on a background of 64% expressing general financial concerns. These resources were widely used, considered essential for success in summative assessments, and perceived as a cultural norm among medical students. Students also acutely recognised the inequities that use of these commercial resources created within their peer group.

**Conclusions:**

Medical schools should recognise the ubiquitous use of paid-for commercial resources among their students and the consequent inequities, which may disproportionately impact individuals from disadvantaged backgrounds. We recommend medical schools provide their students with guidance on the potential benefits and pitfalls of commercial resources, including the development of skills to appraise content critically. Additionally, institutions should explore strategies to mitigate disparities in access, ensuring that all students, irrespective of their socioeconomic status, are afforded equitable opportunities to succeed.

**Supplementary Information:**

The online version contains supplementary material available at 10.1186/s12909-025-07233-4.

## Background

Student engagement and self-regulated learning (SRL) encompasses cognitive, behavioural, motivational and emotional aspects of learning [1]. The motivational drivers of students’ self-regulated learning can be considered using self-determination theory, which distinguishes between intrinsic and extrinsic motivators [2]. Intrinsic motivation, characterised by the highest level of self-regulation, stems from innate interest or enjoyment, whereas extrinsic motivation is driven by the pursuit of specific outcomes such as financial reward and accolades. In undergraduate medical education, formal curricula are typically designed to foster intrinsic motivation, encouraging students to engage in learning activities that prepare them for future clinical practice and develop core competencies required for early postgraduate training. However, these intrinsic motivators are often overshadowed by hidden curricular effects [3] wherein extrinsic motivators dominate students’ focus on short-term goals, particularly immediate success in medical school examinations [4]. This emphasis on extrinsic motivation arises from students’ aspirations to progress academically, improve year rankings, secure prizes, and achieve higher degree classifications– all of which play a significant role in recruitment processes within a highly competitive job market [5]. Consequently, extrinsic motivators drive intense competition among medical students, leading to assessment-driven learning behaviours aimed at outperforming peers [6].

Medical students, driven by the pursuit of higher examination performance, therefore utilise a wide range of learning opportunities, whether those resources are provided by the medical school or by third-party commercial organisations. A growing global trend is the increasing use of paid-for commercial resources, such as question banks and virtual patient scenarios, which supplement medical school-provided materials by offering additional learning and assessment material [7]. Research has demonstrated that medical students find question banks to be a time-efficient learning strategy, particularly before summative examinations, citing benefits such as immediate scoring, explanatory feedback, and consolidation of learning outcomes [8, 9]. In the UK, the proliferation of paid revision platforms has been fuelled by the introduction of the national Medical Licensing Assessment (MLA), which are marketed as helping students to succeed in high-stakes assessments. Research from the United States Medical Licensing Examination highlights the ubiquitous use of commercial resources by candidates, driven by a perceived necessity and their association with improved board exam performance [9]. Further research from Australia highlights the popularity of question banks for revision, though concerns around curriculum alignment were highlighted [10]. A recent qualitative study from a UK medical school further revealed that student engagement with question banks was primarily extrinsically motivated, driven by the desire to excel in summative assessments and the fear of examination failure without these resources [11]. However, this study’s scope was limited to ten early-year medical students.

Although emerging evidence highlights medical students’ increasing reliance on commercial learning resources, little attention has been given to the inequities these resources may generate due to their paid subscription models. This issue is particularly significant in the context of Widening Participation (WP) in UK-based medical education. WP refers to a process by which students from traditionally underrepresented backgrounds, including those defined by socioeconomic status, ethnicity, gender, age, or disability are encouraged to enrol and succeed in higher education [12]. Whilst the total cost of being able to successfully complete an undergraduate medical degree is ever increasing (including tuition fees and accommodation), WP students are disproportionately affected by the financial demands placed upon them, with an estimated potential annual shortfall of £3248 for UK medical students from the lowest income households [13]. The British Medical Association’s 2022 student survey revealed widespread financial hardship, with many students applying for hardship funding and over half taking on term-time employment [14]. Alarmingly, over 60% of the survey respondents reported reducing spending on essentials, 3.9% resorted to food banks, and 44.3% expressed concern about running out of money before the end of the academic year. Bergmann et al. (2019) highlighted the additional stress faced by medical students juggling part-time jobs with their studies, underscoring the cumulative impact of financial pressures on their academic and personal well-being [9].

Paid-for commercial resources must therefore be considered within the broader context of medical students’ financial challenges. Understanding these issues is crucial to fostering a more inclusive and equitable medical education system. This study sought to explore these issues through the following research questions:

1. What is the current landscape across UK medical schools regarding the provision and guidance on medical students’ use of paid-for commercial educational resources?
2. What are the views of medical students at a large UK medical school on the use of paid-for commercial educational resources and their implications for equity of access?

## Methods

To address the first research question, a brief electronic Qualtrics^®^ questionnaire was sent to the heads of assessment of 43 UK medical schools (the questionnaire was developed specifically for this study and is provided in Appendix 1). Respondents were asked to indicate (a) if their school provided guidance for their students regarding paid-for commercial revision platforms and (b) if they offered funding for their students to access such resources. This component of the study was reviewed by the University research ethics team and deemed exempt from formal ethical review (Ref 2023-17980-30589). Responses were analysed and presented descriptively.

The second research question was explored using another Qualtrics^®^ questionnaire sent to all Year 3 and Year 4 medical students at our own institution, the largest medical school in the UK (the questionnaire was developed for this study and is provided in Appendix 2). Respondents were asked to indicate their typical annual spend on paid-for commercial resources, the frequency of their use, the perceived impact on their subsequent examination outcomes and their views and attitudes to their implementation within their studies. Additionally, the questionnaire explored the prevalence of student financial concerns including asking students if they had been recipients of free school meals at secondary school, which is a recognised marker for financial hardship [15]. Questions were drafted in light of the known existing literature base, its gaps and the research questions set. Although the survey was not piloted with students prior to distribution, it was co-created with the lead author who was a medical student at the time. Recruitment for the study was facilitated through advertisements posted on the University’s medical student society social media pages. Participation was entirely voluntary, with responses anonymised as outlined in the participant information sheet. The student questionnaire included both qualitative free-text response fields and Likert-type rating scales. Due to the potential ethical sensitivities associated with the inequity issues explored in this questionnaire, a formal ethics board review was required and subsequently approved (Ref 2021-12924-20739). Quantitative results from the questionnaire were analysed descriptively by the full cohort and for each of the WP and non-WP groups. Due to the small sample size, inferential statistics were not conducted. Qualitative free-text comments were analysed inductively by two of the study authors for recurring concepts that were later grouped into key themes in line with the stages of reflective thematic analysis [16]. In accordance with this approach, the use of dual coders was to enrich an interpretivist data analysis approach rather than to achieve a positivist-perspective consensus / inter-rater reliability.

## Results

### Medical school questionnaire

Thirty of the 43 (70%) surveyed medical schools responded to the survey. Half of the respondents indicated they offered guidance to their students about the use of paid-for commercial revision/assessment platforms (Table 1). Free-text comments revealed that this advice varied with some encouraging their students to primarily use revision materials provided by the school or peer teaching society, and others highlighting that the use of paid resources should be viewed as optional and not necessary to succeed on the course. Other schools advised students that they were unable to endorse any particular paid-for resources and instead directed students to freely available learning resources. Assessment leads expressed concerns about the robustness of quality assurance mechanisms in these platforms. Specifically, they questioned whether the materials provided were clinically accurate, regularly updated, and adequately aligned with the learning objectives and assessment methodologies employed within their institutions.

**Table:** Table 1

Does your medical school offer guidance to your students about the use of paid-for/commercial revision or assessment platforms?	
No	15
Yes	15

Medical schools were also asked to indicate if they offered any funding or access for their students to use for paid-for commercial resources (Table 2). Five schools did not complete this question. Seven of the remaining 25 schools (28%) indicated they did provide funded access to these systems. Free-text comments from these seven schools revealed that six provided licences for students to use platforms that were centrally selected and funded by each school

**Table:** Table 2

Does your medical school offer any funding or access for students to access paid-for/commercial revision or assessment platforms?	
No	18
Yes	7

### Medical student questionnaire

Ninety-nine students out of 915 (10.8%) responded to the student questionnaire, of which 36% were in Year 3 and 64% were in Year 4 of the medicine programme. Students from a widening participation background constituted 12% of the respondent group. Other baseline demographics are provided in Table 3.

**Table 3:** Respondent demographics

Characteristic	Total number of respondents, *N* = 99	Percentage of the total respondents (%)
**Year**		
Year 3	36	36.4
Year 4	63	63.6
**Gender**		
Male	42	42.4
Female	57	57.6
**Widening Participation students**	12	12.1
**Free school meal recipients**	10	10.1
**Bursary recipients**	25	25.3
**Amount of bursary**		
£1,000	12	12.1
£2,000	15	15.2
£4,000	3	3.0
N/A	69	69.7
Abbreviations: N, number of participants, NA not applicable		

**Fig. 1:**
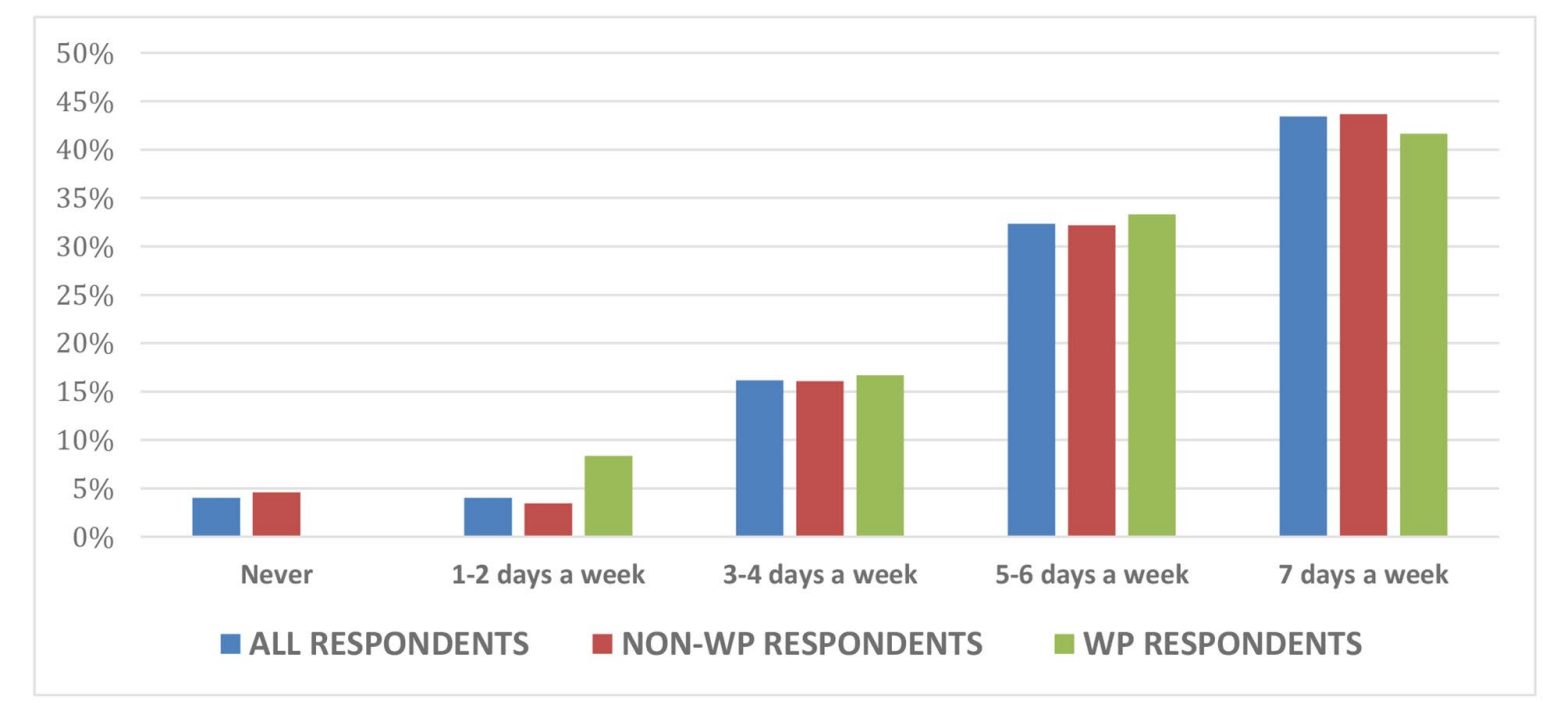
Frequency of medical students’ use of paid resources leading up to examination

**Fig. 2:**
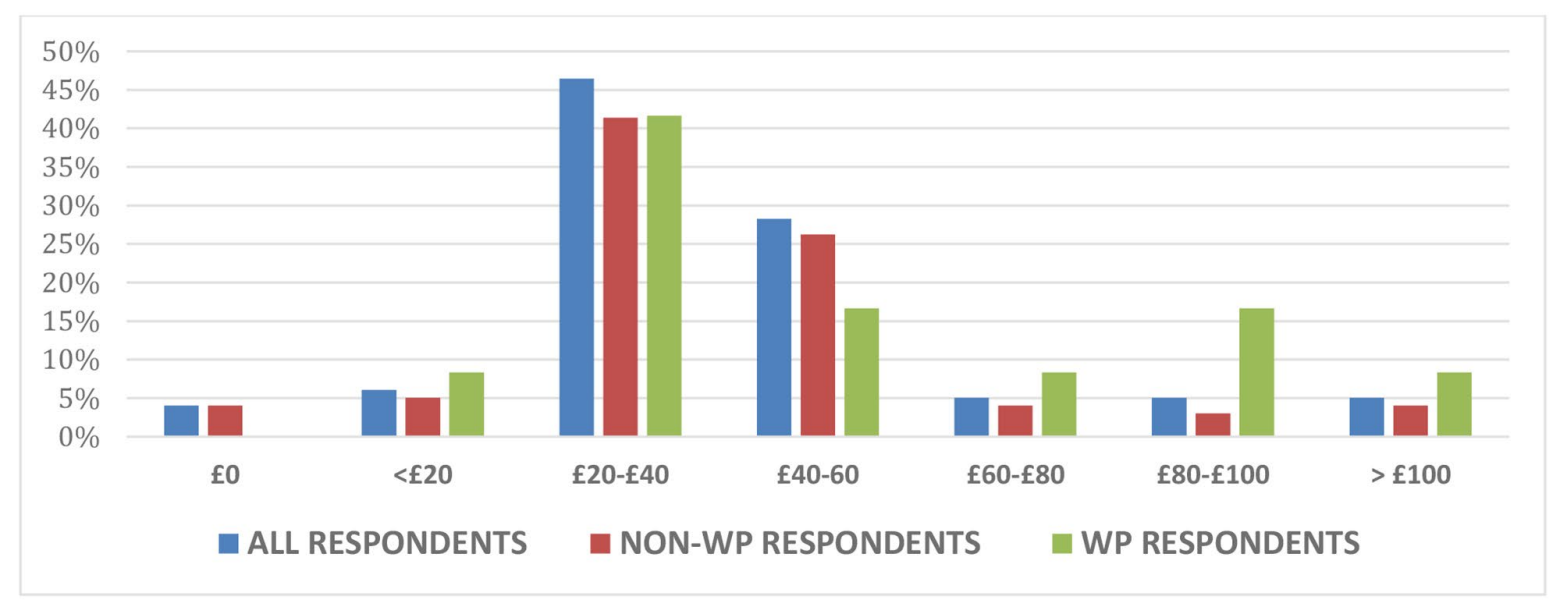
Proportion of payments made by medical student participants towards resources per annum

**Fig. 3:**
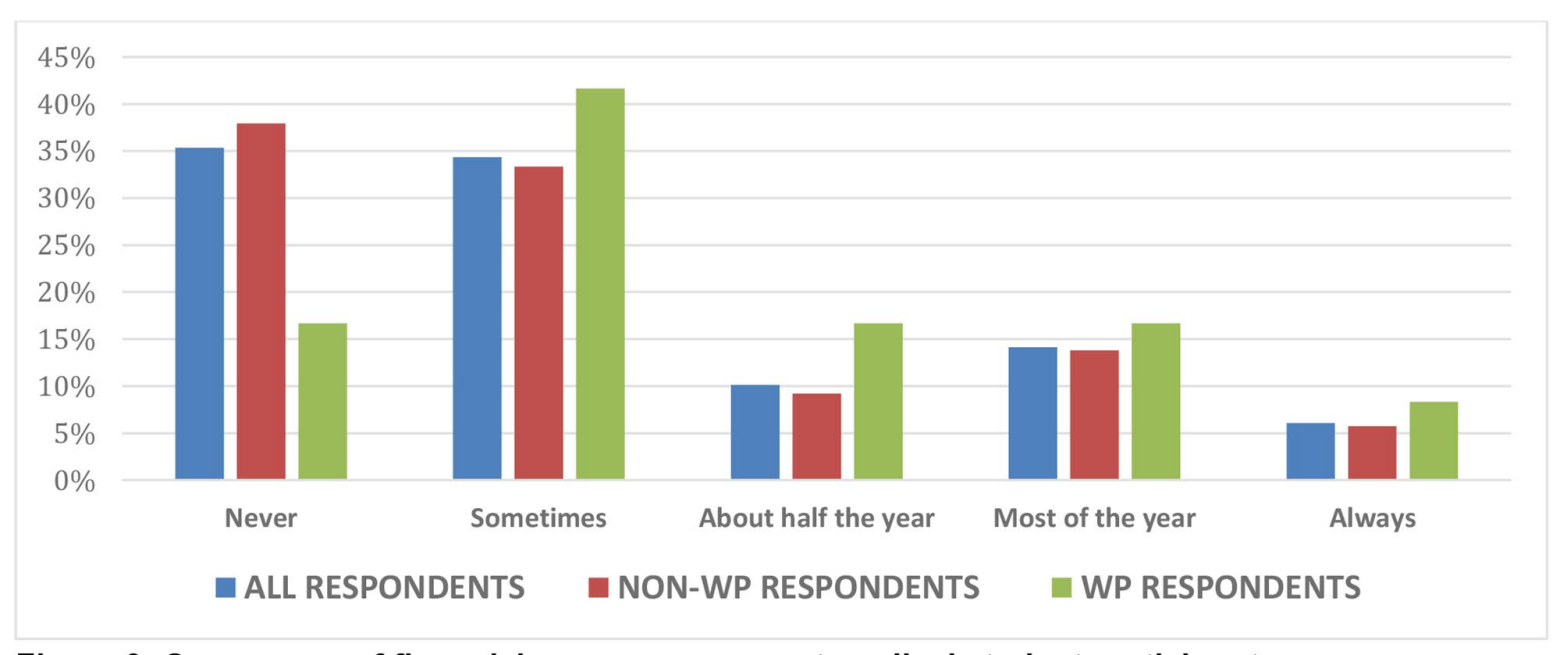
Occurrence of financial concerns amongst medical student participants

The majority of respondents (95%) preferred using paid-for commercial resources over university-provided resources in their revision for forthcoming summative examinations. Of the full respondent cohort, only 4% reported they had never used paid resources, with the remaining respondents indicating a range of frequency of use up to seven days a week (Fig. 1). These usage figures were comparable for both WP and non-WP students. Payments made towards commercial resources varied with 47% of full respondent cohort paying between £20-£40 per academic year, and 6% paying upwards of £100 per annum (Fig. 2). Again, this spread was similar across the WP and non-WP students. With regard to potential inequity of access, 12% of students indicated that they had been recipients of free school meals at secondary school, an aforementioned marker for financial hardship. However, 65% of all respondents were currently facing some degree of financial concerns, with 83% of WP students vs. 62% of non-WP students citing concerns about their finances (Fig. 3).

The free-text analysis generated three key themes: [1] Paid resources are perceived by students as being essential for success; [2] Using paid resources is seen as the norm; and [3] Accessing paid resources creates inequalities among students.

### Theme 1: paid resources are perceived by students as being essential for success

Medical students regarded paid resources as essential for academic success, with many reporting that these tools had a positive impact on their examination preparation and overall academic performance.

Participants reported that signing up for paid resources was “*completely necessary for progression*” (Non-WP, Year 3) and “*crucial part of my revision*” (Non-WP, Year 4). Students feared that not using these resources would result in them struggling to perform well in their examinations: “*Paid online resources have become a necessity in med school*,* and without them*,* I truly do not think I would feel equipped enough to even achieve a ‘satisfactory’ in my [next assessment]*” (Non-WP, Year 3).

Participants reported that the main advantage of using paid resources compared to other resources available to them was the quick, easy access to large question banks that they felt were representative of their subsequent exam content: *“The access to a bank of practice questions trumps other resources as they best replicate the exam scenarios”* (WP, Year 4). Students highlighted that question bank content seemed to be relevant and up-to-date and allowed coverage of both breadth and depth of learning with repeated deliberate practice: *“The amount of practise questions and very clear explanations makes learning very easy. It is also quite satisfying watching yourself progress as you get more and more questions right” (Non-WP*,* Year 4).* The positive feedback offered by these platforms, in turn, positively impacted students’ readiness as examinations drew closer: *“[their use] hugely improved my confidence going into the progress test”* (Non-WP, Year 3).

### Theme 2: using paid resources is seen as the norm

The reliance upon paid resources was evident for most of the participants in both responses to the quantitative frequency of use question reported previously and additional free-text comments, including: *“most people that I know are subscribed to at least one paid resource” (Non- WP*,* Year 3)* and *“everyone uses them”* (Non- WP, Year 4). This therefore created a hidden culture of peer pressure to sign up for commercial platforms for fear of not being on an equal footing with their fellow students: *“I use the free versions or free trials of paid resources*,* which are really useful for learning*,* but I feel like I am missing out by not using the full paid versions”* (Non-WP, Year 4).

The use of paid resources was a well-established and accepted learning route with recommendations for their use arising from more senior students or newly qualified doctors: *“Students in the year above / doctors always recommend using [redacted paid platform] as standard to do well in exams” (Non-WP*,* Year 4).* Beyond their perceived positive impact on assessment outcomes, these resources were also seen to offer efficient ways to maximise learning, particularly for students who had other commitments alongside their undergraduate studies, such as employment: *“It is a really efficient way to use my time*,* especially because I work part-time”* (WP, Year 4).

### Theme 3: Accessing paid resources creates inequalities among students

Whilst the use of paid resources was prevalent, those who did not have the financial means to access these instead opted to use freely available online resources. Some participants, from both a WP background and non-WP background, expressed that their difficulties in being able to self-fund these learning resources created upset and additional stress for students who were already struggling: *“Students casually talk about using paid resources*,* which can be really alienating at a time when I can’t afford them”* (WP, Year 4), *“I think that question banks are beneficial tools for revision*,* but I resent having to pay for them”* (Non-WP, Year 4).

These feelings were compounded by reported unfair academic advantage for those who were able to fund access to one or indeed several paid resource platforms versus those who could not:

*“Paying extra for resources on top of maintenance costs is overwhelming*,* especially for students from less privileged backgrounds. It’s disappointing that our more privileged peers may have an advantage when it comes to [our exams] as they are able to finance [access to] paid resources”* (WP, Year 4).

“*It feels frustrating that personal financial situation is a barrier to resources that may put students at an advantage”* (Non-WP, Year 4).

Additionally, participants expressed frustration that their use of paid resources was typically left to their own judgment, with little or no formal guidance provided to help students decide which, if any, of the commercially available systems they should sign up for. This was particularly pertinent to WP students for whom efficient, targeted use of funds was paramount: “*Since there are so many resources and the university doesn’t advertise which ones are helpful*,* students like me end up spending money that we may not need to*,* or miss out on valuable resources”* (WP, Year 4).

As a result of these financial concerns, many students expressed a desire for specific medical school funding to create equity of access and equity of learning experience: *“It would be good if the medical school could give us a bursary/allowance for this as every student learns differently”* (Non-WP, Year 4). *“I think medical schools need to provide these resources to their students as their resources are not keeping up with the modern world and increasing competition in exams” (WP*,* Year 4)*.

## Discussion

The study uniquely explores the approaches of medical schools and the perspectives of medical students regarding paid commercial educational resources, with a particular focus on potential disparities in access. This issue has gained increasing significance following the introduction of the UK Medical Licensing Assessment (UKMLA) and a subsequent rapidly expanding market of subscription-based commercial platforms targeting medical students. Our findings highlight a nationally inconsistent approach among medical schools with half of respondents providing their students with guidance on paid resources, and seven offering specific funding support. Within our own institution, 96% of student respondents were paying for commercial resources, driven by the perceived link between their use and improved assessment outcomes. However, this should be considered in the context of 65% reporting facing financial concerns; a figure that rises to 83% for those from a WP background. Interestingly, despite these differences, WP and non-WP students reported similar expenditure on commercial resources and comparable frequency of use. This lack of difference may be attributed to the financial support provided by our institution for WP students as explained later. Furthermore, students expressed a desire for more formal support from the medical school to address potential disparities of access.

Our findings align with existing literature highlighting how assessment-driven learning behaviours dominate medical students’ approaches to education. Medical students are increasingly motivated by external rewards, a well-documented phenomenon in medical education, where assessment plays a powerful role in steering learning [17]. Peer pressure also contributes to this dynamic, as evidenced by qualitative responses in our study. Students report that reliance on paid resources has become culturally normalised, and those unable to afford these resources feel disadvantaged and pressured to conform. This mirrors findings from other contexts, where the hidden curriculum influences student behaviour and fosters competition among peers [3]. In our study, senior students and recent graduates were found to recommend the use of these resources, perpetuating a cycle of reliance on commercial platforms for academic success. Additionally, contextual literature indicates that students who perform well tend to be more socially connected to other high-performing students [18]. Extrapolating from this, informal learning networks in medical schools, including the sharing of external resources such as question banks, may play a crucial role in further reinforcing the hidden curriculum. Exploring this dynamic further could shed light on its impact, particularly on medical students from WP backgrounds.

The psychological impact of resources to prepare for examinations should not be understated. In our study, it was highlighted that some students felt that paid resources were a ‘necessity’ and had significant impact on their confidence levels before examinations. In a USA context Bauzon et al. found that students who felt more prepared for exams performed better than those who felt less prepared [19]. However, the authors also reported that students who purchased commercial resources did not significantly improve their exam performance; instead, didactic lectures and peer-to-peer teaching were found to offer more substantial benefits. This suggests that while paid resources may boost students’ confidence, their direct impact on academic outcomes remains uncertain. Further research exploring the psychological impact of various commercial resources on the preparedness and performance of medical students in the UK would provide valuable insights into this complex issue.

Notably, no respondents in our study expressed concerns about the quality of the learning material or question items they encountered from commercial resources, despite these concerns being raised by medical schools. Commercial resources are not subject to the same rigorous quality assurance processes as accredited undergraduate programs, which raises the risk of inadvertently promoting suboptimal or incorrect clinical practices. Furthermore, these resources may not fully align with formal curricular objectives, potentially leading to confusion among learners already grappling with high levels of assessment-related anxiety [9] This highlights the need for careful consideration of the quality and alignment of commercial resources with medical curricula to ensure they support, rather than hinder, student learning and how best students are supported to critically review system content.

A recent UK study examining medical students’ use of practice questions found that they used them both for revision and as a primary learning tool, highlighting the importance of answer rationales [8]. While this research highlighted why question banks are used, our study aimed to delve deeper into the frequency of their use and the equity concerns they raise. A key issue highlighted in our findings is the financial burden that paid resources place on medical students, particularly those from Widening Participation (WP) backgrounds. With medical education already costly, additional expenditures on educational tools exacerbate existing financial strains. In our study, 64% of students reported facing financial concerns, a finding consistent with national surveys like the British Medical Association’s 2022 report, which revealed that many medical students had to cut back on essentials, rely on food banks, or work part-time to meet their financial needs [14]. This financial stress not only impacts students’ well-being but may also hinder their academic performance, as time spent working detracts from study time [9].

These financial barriers are particularly pronounced for WP students, who already face systemic disadvantages in higher education [12]. The fact that paid resources are perceived as essential by most students only deepens the inequalities between those who can afford these resources and those who cannot. This situation raises significant concerns about the fairness of the current system, where financial means may directly influence academic outcomes. Such a disparity undermines efforts to widen participation in medicine and to create a level playing field for all students, highlighting the need for more equitable solutions in medical education.

While this study offers valuable insights, several limitations must be acknowledged. The survey achieved a response rate of 99 students which formed 10% of the total invited cohort and as such, respondents may not fully represent the wider medical student population. Notably, the sample size for WP students (12.1%) was relatively small and underrepresented compared to the annual cohort demographics, where WP students are estimated to comprise around 22%. This may limit the generalisability of findings, particularly concerning financial inequities. Second, we recognise that the survey was not validated through a formal pilot / think-aloud protocol. However, it was designed jointly with the lead author who was a medical student at the time this study was conducted and with most questions requiring a factual response minimising the potential for participant misinterpretation. The study was conducted at a single UK-based institution, and we recognise that the experiences of medical students at other universities may differ. The findings however are likely to be similar across other schools both within the UK and beyond and thus offer useful generalisability. The qualitative component of the study relied on a single free-text question, which may have restricted the depth of insights. Furthermore, as this question appeared at the end of the survey, participant fatigue may have influenced the quality and quantity of responses. Although we have reported findings split by WP and non-WP cohorts, the small sample size prevented inferential statistical comparisons between groups. Although general financial concerns were assessed, more detailed data on spending across demographic groups would have provided a clearer picture of the inequities faced by WP students. Finally, participation was voluntary, and while financial incentives were avoided to minimise bias, this voluntary approach may have affected engagement and the diversity of responses obtained.

### Recommendations

We recommend medical schools provide their students with guidance on the potential benefits and pitfalls of commercial resources, including the development of skills to appraise content critically. Furthermore, schools may wish to consider offering funded access to these systems, particularly for WP students, to reduce financial barriers and promote equitable opportunities for success. At our institution, as of 2024, free access to a third-party commercial resource is offered to all medical students, alongside an ongoing annual bursary for lower-income students [20]. We encourage other medical schools in the UK review how their students may be impacted by the plethora of available commercial paid resources and to consider similar schemes underpinned by equality impact assessments.

Further research is essential to assess the long-term effects of paid resources on academic outcomes across undergraduate and postgraduate medical training. A multi-institutional longitudinal study could yield more comprehensive insights into how these resources are utilised and the financial challenges they pose to students nationwide. Additionally, future studies should explore the influence of peer pressure and the hidden curriculum in shaping students’ reliance on commercial educational resources, with in-depth qualitative interviews providing valuable perspectives. Research should also investigate how medical students can be best supported in critically evaluating commercial resources, addressing concerns around quality assurance, clinical accuracy, and alignment with formal curricula.

## Conclusions

While paid-for commercial resources are widely regarded as valuable tools for exam preparation and are extensively used by medical students, their financial cost presents significant challenges, particularly for WP students. As reliance on these resources continues to grow, we recommend that medical schools adopt proactive measures to address these inequities. These efforts should extend beyond financial support to also help students navigate, select, and critically evaluate the increasing array of commercial platforms available.

## Electronic supplementary material

Below is the link to the electronic supplementary material.

Supplementary Material 1

Supplementary Material 2

## Data Availability

The datasets used and/or analysed during the current study are available from the corresponding author on reasonable request.

## Abbreviations

MLA: Medical Licensing Assessment
SRL: Self regulated Learning
USMLE: United States Medical Licensing Examination
WP: Widening Participation
