# Supplementary material for: Pay to win? Exploring medical students’ use of, and access to, paid commercial educational resources
Source: BMC Med Educ. 2025 May 21;25:738. doi: 10.1186/s12909-025-07233-4 (PMC12093721; doi:10.1186/s12909-025-07233-4)
Supplement: Supplementary file 1 — Supplementary Material 1 [file 12909_2025_7233_MOESM1_ESM.docx]

**APPENDIX 1:**

**Questionnaire for UK Medical Schools**

1. Does your medical school offer guidance to your students about the use of paid-for/commerical revision or assessment platforms

Yes

No

1. If Yes: Please summarise what guidance you offer
2. Does your medical school offer any funding for students to access paid-for/commerical revision or assessment platforms?

Yes

No

1. If Yes: Please describe the funding arrangement in place including amount per student (if willing to share this), any particular target groups of students/ eligibility criteria and any criteria regarding usage
2. Any additional comments:
