# Supplementary material for: Pay to win? Exploring medical students’ use of, and access to, paid commercial educational resources
Source: BMC Med Educ. 2025 May 21;25:738. doi: 10.1186/s12909-025-07233-4 (PMC12093721; doi:10.1186/s12909-025-07233-4)
Supplement: Supplementary file 2 — Supplementary Material 2 [file 12909_2025_7233_MOESM2_ESM.docx]

**APPENDIX 2:**

**Questionnaire for medical students

Demographics** 
 
Before we ask our questions, we would be grateful if you could tell us a bit more about yourself. As explained, all details will remain anonymous:

Demographics What year of the MB ChB programme in Manchester were you in the academic year 2020-2021

- Year 3 (1)
- Year 4 (2)

Demographics To which gender identity do you most identify?

- Male (1)
- Female (2)
- Non-binary / third gender (3)
- Prefer not to say (4)

Demographics Which ethnicity would you use to describe yourself?

- White; English, Welsh, Scottish, Northern Irish or British (1)
- White; Irish (2)
- White; Gypsy or Irish Traveller (3)
- White; Any other white background (4)
- Mixed or multiple ethnic backgrounds; White and Black Caribbean (5)
- Mixed or multiple ethnic backgrounds; White and Black African (6)
- Mixed or multiple ethnic backgrounds; White and Asian (7)
- Mixed or multiple ethnic backgrounds; Any other Mixed or Multiple ethnic background (8)
- Asian or Asian British; Indian (9)
- Asian or Asian British; Pakistani (10)
- Asian or Asian British; Chinese (11)
- Asian or Asian British; Bangladeshi (12)
- Asian or Asian British; Any other Asian background (13)
- Black, African, Caribbean or Black British; African (14)
- Black, African, Caribbean or Black British; Caribbean (15)
- Black, African, Caribbean or Black British; Any other Black, African or Caribbean background (16)
- Arabic (17)
- Any other ethnic group (18)

Demographics What type of student are you?

- UK Undergraduate (1)
- UK Postgraduate (2)
- International student (3)
- EU student (4)

Demographics Did you intercalate before the academic year 2020-2021?

- Yes (1)
- No (2)

Demographics Do you identify as a Widening participation student? (Did you attend an underperforming school, be in care, live in a deprived postcode at the time of applying to medical school, received a contextual offer for Medicine at University of Manchester, such as through Manchester Access Programme)

- Yes (1)
- No (2)

Demographics Have you ever received free school meals?

- Yes (1)
- No (2)

| Page Break |  |
| --- | --- |

Q16 **Finances**  
 
We would also like to ask you some questions about your financial situation. All information will remain anonymous. These questions will utilise likert scales.

Finances Financial situation

|  | Never (1) | Sometimes (2) | About half the year (3) | Most of the year (4) | Always (5) |
| --- | --- | --- | --- | --- | --- |
| During the academic year of 2020-21, have you been worried about your financial situation? (1) |  |  |  |  |  |

Finances How have you funded your degree (tuition and living costs) DURING 2020-2021?

- Student loan company (1)
- Inheritance (2)
- Savings (3)
- Parental support (4)
- Summer/winter holiday paid work (5)
- Term time paid work (6)
- NHS bursary (7)
- Bursaries from the university/other organisations (NOT NHS) (8)
- Bank loan (9)
- Other (10)

Finances Did you receive the Manchester Bursary DURING 2020-2021? (this is available to any UK student who is registered on an eligible undergraduate degree course here and who has had a full financial assessment carried out by Student Finance. The household income is assessed and if below £35,000 the student will receive an award)

- Yes (1)
- No (2)

| Page Break |  |
| --- | --- |

Finances If you have answered yes to the previous question, how much money did you receive?

- £1000 (4)
- £2000 (5)
- £4000 (6)

| Page Break |  |
| --- | --- |

Study resources **Study Resources**
 
We would now like you to answer questions around resources used for your studies.

Study resources Resource expenditure (if the amount falls exactly between 2 categories e.g £40, please round upwards)

|  | £0 (1) | < £20 (2) | £20-£40 (3) | £40-60 (4) | £60-£80 (5) | £80-£100 (6) | > £100 (7) |
| --- | --- | --- | --- | --- | --- | --- | --- |
| During your 2020-2021 year at medical school, how much have you spent in total on paid online resources? (1) |  |  |  |  |  |  |  |

Study resources Use of paid online resources

|  | Never (1) | 1-2 days a week (2) | 3-4 days a week (3) | 5-6 days a week (4) | 7 days a week (5) |
| --- | --- | --- | --- | --- | --- |
| In the lead up to the progress test, how often are you using paid online resources for revision in a week? (1) |  |  |  |  |  |

Study resources Use of university online resources

|  | Never (1) | 1-2 days a week (2) | 3-4 days a week (3) | 5-6 days a week (4) | 7 days a week (5) |
| --- | --- | --- | --- | --- | --- |
| In the lead up to the progress test, how often are you using university online resources for revision in a week? (1) |  |  |  |  |  |

Study resources In the lead up to the progress test, if you could ONLY use ONE resource which would you choose?

- Paid online resources (1)
- University online resources (2)

| Page Break |  |
| --- | --- |

Q26 **Progress Test Performance**
 
We would now like to explore your achievement in the 20/21 progress tests

Q27 What grade did you achieve in your **JANUARY** 2021 progress test?

- Unsatisfactory (1)
- Low pass (2)
- Satisfactory (3)
- Honours (4)
- Distinction (5)

Q28 What grade did you achieve in your **MAY** 2021 progress test?

- Unsatisfactory (1)
- Low pass (2)
- Satisfactory (3)
- Honours (4)
- Distinction (5)

| Page Break |  |
| --- | --- |

Q29 **Your view on paid online resources**

Finally, we would like to gauge your views on paid online resources

Q30 How do you feel about paid online resources and the impact, if any, of these on medical school performance?
